# The Association Between Leisure Games and Cognitive Functioning Among Older Adults

**DOI:** 10.1093/geroni/igab046.2616

**Published:** 2021-12-17

**Authors:** Tyran Terada, Yeonjung Lee

**Affiliations:** 1 University of Hawai`i, Honolulu, Hawaii, United States; 2 University of Hawaiʻi at Mānoa, Honolulu, Hawaii, United States

## Abstract

Stimulating leisure games have been shown to offer cognitive stimulation among older adults. This cross-sectional study examined the association between word games (crossword puzzles and scrabble) / cards and games (games such as chess) and cognitive functioning among adults aged 65 years and older (n=3271). All data were collected from the Health and Retirement Study (2016). Results from the hierarchical regression models suggest that higher levels of participation in word games (p<.01) and cards and games (p<.01) predicted higher levels of cognitive functioning. In the final model, after controlling for age, gender, ethnicity, marital status, education, and income, a total variance of 31 percent was explained. All covariates were statistically significant except marital status. Word games (β=.117, p<01) and cards and games (β=.054, p<.01) had a significantly positive association with cognitive functioning. These findings suggest that participation in word games and cards and games are associated with cognitive functioning among older adults.

